# Prolonging the Life Span of Membrane in Submerged MBR by the Application of Different Anti-Biofouling Techniques

**DOI:** 10.3390/membranes13020217

**Published:** 2023-02-09

**Authors:** Noman Sohail, Ramona Riedel, Bogdan Dorneanu, Harvey Arellano-Garcia

**Affiliations:** 1 Department of Biotechnology of Water Treatment, Brandenburg University of Technology Cottbus/Senftenberg, 03046 Cottbus, Germany; 2 Department of Process and Plant Technology, Brandenburg University of Technology Cottbus/Senftenberg, 03046 Cottbus, Germany

**Keywords:** membrane bioreactor (MBR), quorum sensing (QS), quorum quenching (QQ), moving bed biofilm reactor (MBBR), moving bed biofilm membrane reactor (MBBMR), model-based anti-fouling strategies

## Abstract

The membrane bioreactor (MBR) is an efficient technology for the treatment of municipal and industrial wastewater for the last two decades. It is a single stage process with smaller footprints and a higher removal efficiency of organic compounds compared with the conventional activated sludge process. However, the major drawback of the MBR is membrane biofouling which decreases the life span of the membrane and automatically increases the operational cost. This review is exploring different anti-biofouling techniques of the state-of-the-art, i.e., quorum quenching (QQ) and model-based approaches. The former is a relatively recent strategy used to mitigate biofouling. It disrupts the cell-to-cell communication of bacteria responsible for biofouling in the sludge. For example, the two strains of bacteria *Rhodococcus* sp. BH4 and *Pseudomonas putida* are very effective in the disruption of quorum sensing (QS). Thus, they are recognized as useful QQ bacteria. Furthermore, the model-based anti-fouling strategies are also very promising in preventing biofouling at very early stages of initialization. Nevertheless, biofouling is an extremely complex phenomenon and the influence of various parameters whether physical or biological on its development is not completely understood. Advancing digital technologies, combined with novel Big Data analytics and optimization techniques offer great opportunities for creating intelligent systems that can effectively address the challenges of MBR biofouling.

## 1. Introduction

Water is the basic need of life according to Maslow’s hierarchy. It is an essential necessity of life for all living things in the universe as a source for drinking, cleaning, and food production. One third of the planet is covered with water. It is used abundantly for both domestic and industrial purposes. Therefore, it is important that purified water should be available for human consumption. A good source of the drinking water is ground water because of the underground storage and natural purification properties of the soil [1]. The availability of fresh potable water is becoming a hot topic in many Asian countries, very specifically in Pakistan [2].

According to the World Resource Institute, by 2030 Pakistan is going to be among the top 33 countries which will face extreme water scarcity. Pakistan is considered the 14th out of 17 most water-stressed countries in the world. Over 80% of the total population has experienced severe water scarcity for at least one month in a year [3]. The accessibility of water has reduced to 1017 m^3^/person, which is a very startling situation for Pakistan [4]. The pollution of the resources of fresh water, due to the municipal and industrial wastewater discharge, has made the situation even more alarming and threatening [2].

The most important environmental challenge around the world is related to the municipal sewage waste and its treatment. Eighty percent of water-borne diseases in developing countries are caused by sewage. Furthermore, one fourth of the children under the age of five die each day because of this alarming issue. Overall, about 30,000 people die from water related diseases each day. Currently, millions of people suffer from diseases due to poorly treated water. In particular, 400 million people suffer from gastroenteritis related to contaminated water. In addition, 200 million suffer from schistosomiasis, 160 million from malaria, and nearly 30 million from onchocerciasis. All the resources of water are contaminated with agricultural and industrial wastes having toxic organic and inorganic pathogens [1].

Municipal wastewater usually comes along with very high loads of organic compounds and other nutrients such as nitrogen and phosphorus. They can cause severe threats to aquatic environments such as oxygen depletion, eutrophication, and, in some circumstances, high toxicity due to the discharge of hazards and persistent substances. Biological processes such as the removal of organic carbon provide excellent wastewater treatment, especially for municipal wastewater. Other than that, membranes can be used as physical separators [5]. In the field of wastewater treatment, the membrane bioreactor (MBR) is an innovative treatment technology with several advantages, such as a high effluent quality and organic loading rates, smaller footprint, reduced sludge production, improved nitrification/denitrification performance, complete separation of hydraulic retention time (HRT) from the solids retention time (SRT), and easy automatic control [6,7,8]. MBR has been prioritized during the last two decades over the conventional activated sludge process (CASP) due to its high removal efficiency. It is expected that water recycling applications and increased safe water regulations will further lead to an increased application of MBR within the next decades [2].

**Figure 1:**
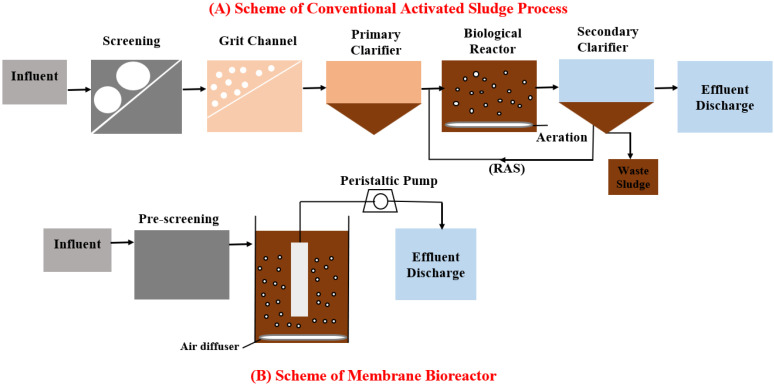
The scheme of the conventional activated sludge process (**A**) and the MBR process (**B**).

MBR technology can remove up to 95% of easily biodegradable organic compounds and 98% of ammonia [9]. This implies that high quality municipal wastewater can be treated using MBRs. Briefly, the MBR replaces the primary and secondary clarifier in the wastewater treatment system (Figure 1). MBR can maintain mixed liquor suspended solids (MLSS) between 8 and 12 g L^−1^ (up to 25 g L^−1^), while CASP commonly operates at 2 to 4 g L^−1^ due to difficulties in settling higher MLSS concentrations in the secondary clarifier [10]. In addition, MBRs are operating at higher MLSS concentrations, longer solid retention times, and less sludge production which avoids the problems of sludge bulking [5].

Despite all these advantages, the major drawback of the MBR remains membrane biofouling, which decreases the life span of the membrane and automatically increases the operational cost. Biofouling associated with MBR is caused by microorganisms and currently little is understood about its key inducing factors. During the fouling process, microorganisms attach on the surface of the membrane and its pores start clogging, and in time this can lead to the formation of cake layers. Furthermore, it was often hypothesised that microorganisms in the MBR might interact together by quorum sensing (QS) [11]. The latter is a phenomenon in which bacteria carry out cell-to-cell communication. This can be a major reason for biofilm formation on the surface of membranes, i.e., biofouling.

Different strategies have been used to control biofouling, including for example, membrane relaxation, standard backwash (SBW) and chemical backwash (CBW), air scouring, or media (plastic and sponge carriers) and quorum quenching (QQ) [12,13,14,15]. SBW is a technique in which the permeate is pumped in the opposite direction of the membrane. Due to the negative pressure, water moves across the membrane and loosely bound particles detach from the surface of the membrane, reducing biofouling [12]. Similarly, strong acids such as sodium hypochlorite can be used in different concentrations for chemical backwashing [13].

Aeration is also considered a solution for anti-biofouling because it does not allow foulants to grow on the surface of the membrane. The use of synthetic nutrient solutions in the MBR plays an important role, reducing cake layer formation due to the physical interactions with the membranes [14].

Quorum quenching (QQ) is a new technique which is used nowadays to mitigate biofouling. It is considered that QQ might be one of the most promising and useful methods in disrupting cell-to-cell communication of the bacteria responsible for biofouling in MBR [16].

Thus, the focus of this review is to provide an overview of the state-of-the-art current developments of different anti-biofouling techniques, especially QQ techniques.

Finally, biofouling is an important factor behind the higher operation costs of the MBRs due to the need for frequent shut-downs for cleaning of the foulants and/or replacements of the membrane [17]. To provide more cost-effective operating and cleaning strategies, the development of model-based predictive solutions of the MBR process is an important area in the past few years [18]. Perspectives on utilizing new developments in the area of process modelling, simulation, and optimization for improved control of biofouling will be briefly discussed.

## 2. Formation of Biofouling in MBR

### 2.1. Brief Overview on the Mechanism of Membrane Fouling

**Figure 2:**
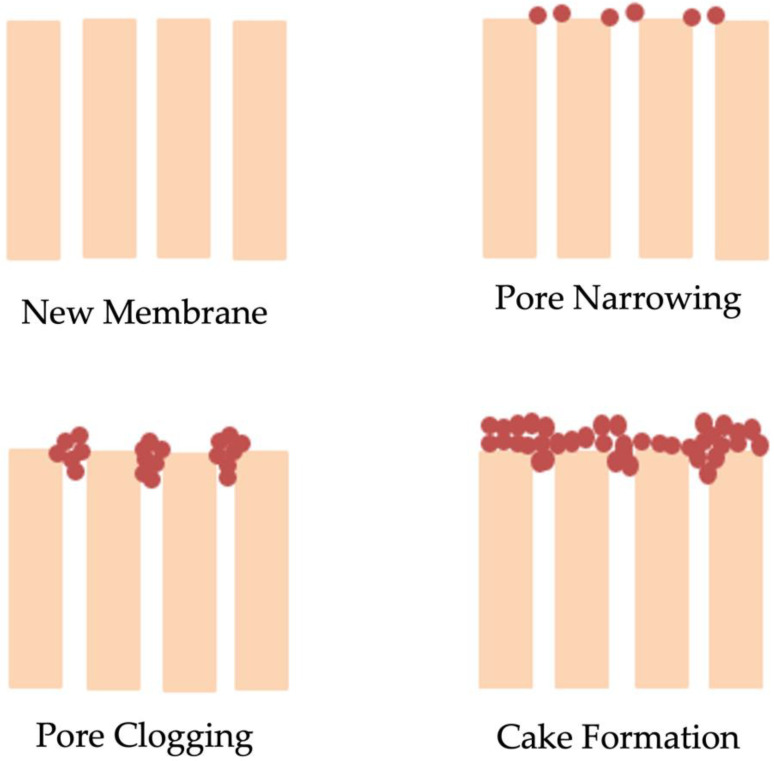
The scheme of the mechanisms of membrane fouling.

During the process of wastewater treatment, particles, as well as colloidal and dissolved matter deposited on the membranes significantly reduce their effectiveness, a process which is known as membrane fouling. These particles are assorted in nature and can be suspended, dissolved, or active microorganisms and form a big part of the MLSS particles (i.e., bioflocs). Membrane fouling is the biggest challenge of the MBR system, as it results in the narrowing of the pores, clogging, and production of cake (Figure 2). When the membrane pores are blocked by the suspended particles, their clogging results. The main factors that have a major influence on the clogging of the pores are the size of the particles. The presence of sticky substances in the solution unfortunately supports the particles in becoming attached to the pores [19]. When the bacteria colonies, biopolymers, and inorganic matter vigorously build up, they result in cake formation and a bio-cake layer formation. These layers increase the filtration resistance of the membrane [20].

### 2.2. Removable and Irremovable Fouling

There are two unusual constituents of the membrane fouling: removable and irremovable fouling. The freely bound fouling part in the membrane filtration is known as reversible fouling. This type of fouling can be eliminated by using substantial means such as relaxation or backwashing techniques. Whereas, when looking upon the irreversible fouling, a tough adherence in the membranes is observed. This phenomenon is the result of pore blockage, gel layer production, and biofilm production, and can be eradicated using chemical cleaning.

When both types of fouling are managed properly, the operational costs of the cleaning schedule of the membranes can be significantly lowered. This property of the MBR makes it a more viable option in comparison with conventional wastewater treatment plants [21].

### 2.3. Transmembrane Pressure Profile

The finest indicators for membrane fouling are the membrane flux and the transmembrane pressure (TMP). When the TMP increases, there is a clear signal that the membranes are fouled and in order to maintain a specific level of flux, the system should operate at a constant pressure [22]. Internal fouling in the membranes is a result of the resistance during filtration. The wastewater sludge filtration is the cause of external fouling of the layers. 

A sudden boost in the TMP is known as a TMP jump. This measure is a characteristic of the internal as well as external fouling. Furthermore, the TMP jump is associated with various fouling rates in the membranes. It often leads to increased fluxes and fouling rates in the areas of lower fouling. The fouling layer is not the same in the depth and composition, because wastewater sludges are assorted mixtures. When homogenous mixtures are present, a pointed TMP rise occurs. Thus, a fast increase in the TMP can also result in the uniformity associated in the biofouling layer [23].

The TMP jump consists of three phases. The first stage is the initial conditioning fouling, caused by the initial pore blockage and solutes adsorption. In the second stage, a slowly linear and exponential rise is observed in the TMP, determined by the biofilm formation and the membrane pore blockage, whereas in the third stage an abrupt fast rise in the TMP is observed, which is mostly the effect of harsh membrane fouling, changes in the localized flux, and the closure of pores. When the TMP increases, the fluxes go beyond their critical values, increasing the deposition of particles and resulting in changes in the cake layer design. Therefore, bacteria in the internal biofilms die due to oxygen limitations and extracellular polymeric substances (EPS) are released. Thus, after stage three, membrane cleaning is necessary [20].

### 2.4. Brief Overview on Important Types of Foulants

Membrane foulants can be classified based on their chemical and biological characteristics in the following categories: biofoulants, organic foulants, and inorganic foulants. The former, i.e., biofoulants, are strongly related to microbial activity caused by microorganisms. They cause the formation of a biofilm on the membrane surface through deposition, growth, and metabolism. Initially, bacteria cells adhere to the membrane surface and get trapped in its pores. Eventually, the cell will multiply into a cluster of cells that will create a cake layer which reduces the permeability of the membrane [22].

Another important type of foulants is related to the organic foulants, biopolymers such as polysaccharides and proteins, which may cause irreversible biofouling. These foulants are produced in the form of EPS by bacteria during metabolic reactions. The deposition of organic foulants on the membrane surface is more challenging to remove compared to big particles such as sludge floc [20].

Finally, the third important fraction of foulants is related to the inorganic foulants. They commonly occur due to chemical and biological precipitation of inorganic/organic substances. If the concentration of metal ions such as Ca^2+^, Mg^2+^, Fe^3+^, Al^3+^, and the anions CO_3_^2−^, SO_4_^2−^, PO_4_^3−^, and OH^-^ increase on the surface of the membrane, they react producing chemical precipitation which becomes the reason for fouling. Furthermore, inorganic particles are already present in the systems which have the ability to adhere to membrane surfaces or obstruct membrane pores, resulting in inorganic fouling [24].

The tendency of membrane biofouling is still one of the major limitations. Biofouling is mainly caused by bounded and non-bounded extracellular polymeric substances. Their relationship with the soluble microbial products is complex and still debated. In simple terms, the complete retention of bioflocs and soluble particles mentioned above has a series of very complex interactions with the membrane in consequence. The production of EPS by microorganisms leads to blockage onto and into the membrane. This causes the hydraulic resistance to quickly increase whereas the permeate flux decreases. The resistance can be subdivided into reversible (removable by back-flushing) and irreversible (non-removable) resistance. The removable fraction is caused by absorption of the suspended solids onto the membrane surface, cake layer formation onto the membrane layer, and concentration polarization directly in front of the membrane. Non-removable resistance is caused by clogging in the membrane pores. Thus, the fouling mechanism is as stated above a combination of standard blocking, cake filtration, intermediate blocking, and complete blocking, respectively. The interactions and/or overlapping of all these phenomena must be considered.

### 2.5. Factors Affecting Membrane Fouling in MBR

There are many factors which are responsible for the fouling inside the MBR systems. They are classified mainly in three types: characteristics of the membranes, operational conditions, and characteristics of the biomass and feed. Various factors are listed below in Table 1 for each of these categories. An extensive overview of the factors influencing the membrane fouling and their mechanisms is presented in [8].

## 3. Anti-Biofouling Strategies

### 3.1. Physical Methods

To maintain the MBR operation and the flux of the membrane, SBW and relaxation techniques are among the most used physical approaches. During SBW, a reverse flow from the permeate side to the feed side is used to effectively remove loosely bounded particles from the membrane surface and foulants within the pores of the membrane [25,26,27]. In submerged MBRs, a 10 min filtration and 1 min backwashing have been successfully used [9].

In the case of the relaxation approaches, the process filtration is stopped to relieve the pressure generated by the membrane [28]. This technique helps to take measures against concentration polarization which lowers down the production of the reversible fouling layer. However, this process has a very limited effect on the removal of the macro- and micro-particle adsorption, because the particles either block the pores of the membrane or get stuck on them, causing irreversible fouling. When these particles are smaller in size than the membrane pores, they get clogged in the pores and irreversible pore narrowing or blockage occurs [21].

Another physical cleaning method is the air scouring, where aeration intensity plays a crucial role to mitigate biofouling. Due to aeration, microbes get less of a chance to deposit on the membrane surface, which ultimately reduces the clogging and the transmembrane pressure [29]. This technique is used to provide oxygen to the microorganisms in the sludge. Furthermore, it allows foulants to move away from the membrane surface due to the shear force in the mixed liquor, which helps to clean the membrane and increases its life span. Recent studies found a 2.5 L min^−1^ aeration intensity to be the most useful in tackling biofouling [26].

Ultrasonic cleaning has been recently investigated as a new physical technique that can generate phenomena such as acoustic streams, microflows, microjets, and shock waves in heterogeneous solid-liquid systems. These physical events demonstrate a wave produced by unidirectional flow currents, which opens the pores of the membrane by the shear force, drag force, and high-pressure shock. Moreover, ultrasonic radiation can minimize the chances of pore clogging by the agglomeration of small particles [7].

### 3.2. Chemical Methods

Chemical cleaning can be performed internally as well as externally while using organic acids, caustic soda, or sodium hypochlorite. Usually, sodium hypochlorite is used to eliminate organic fouling, while citric acid is used against inorganic fouling. Chemical cleaning can also be performed during normal MBR operation by adding a low concentration of chemicals to the backwash water, a process known as chemically enhanced backwashing. The chemical strategy is effective for irreversible fouling, not easily removed in normal MBR operations with simple standard backwash methods. Nonetheless, a major drawback of these approaches is that the intensive use of chemicals may significantly reduce the life span of the membrane [28].

Another problem related to chemical cleaning is the production of toxic and harmful by-products. Researchers are looking for a more environmentally friendly chemical to clean membranes. Peroxymonosulfate (PMS) has been used as a strong oxidant without chlorine in MBR and it was found that its cleaning efficiency is the same as that of NaClO. Ozone has also been used to reduce membrane fouling, as it results in the increase in the size of the sludge flocs by decreasing the zeta potential value and of the surface hydrophobicity of the flocs. This increases the permeability of the sludge suspension.

While selecting chemical oxidants against membrane fouling, the focus of the investigations is on the oxidants and their influence on the microorganisms and the membrane [19]. As a strong oxidant, the excessive use of NaClO has a negative effect on the floc formation and effluent quality. It also inhibits enzymatic activity and changes the structure of the microbial community [30]. Among these methods, sodium hypochlorite is found to be one of the most effective and broadly adopted solutions. The use of NaClO twice a day with 500 ppm was found to be very useful in terms of the operation duration, membrane cleaning, and bacterial growth [16,31].

### 3.3. Hybrid Methods

Municipal wastewater is treated using biological treatments for the removal of organic pollutants. During the past five years, many novel improvements have been made in the field of wastewater treatment. One of the most effective solutions focuses on the combination of two different anti-biofouling technologies for better results and to minimize the drawbacks of the individual approaches [32]. It has been proved through much research that the addition of the moving bed biofilm reactor (MBBR) in an MBR having specialized media is an accurate and significant choice for enhancing the elimination of the nutrients [9]. The utilization of the media in hybrid MBR can be used as a best substitute to conventional MBR, as it results in the enhancement of the treatment efficiency and the reduction in the membrane fouling, a major challenge, as discussed previously [33].

The combination of both these technologies created the moving bed biofilm membrane reactor (MBBMR) process [9]. This process has the capability of using the best characteristics of both biofilm and solid liquid separation. In this hybrid technology, biofilm immobilized on the surface of carriers minimizes the concentration of the suspended solids and, thereby, can reduce drastically the membrane fouling [34]. When the MBR is combined with the moving bed biofilm reactor it results in a decrease in the membrane fouling. Furthermore, it influences the cake layer production on the membrane. During short-term experiments, the critical flux of the hybrid membrane bioreactor increased by 20%, whereas due to the cake resistance it decreased by 86% [35].

Physical cleaning by sponge carriers was proven to be one of the most useful options for improving the MBR performance by mitigating biofouling as moving carriers physically wash the biofouling layer from the membrane surface. The duration of the operation of the MBBMR with sponge carriers was longer than both the simple MBR and the MBBMR with plastic carriers. Furthermore, it was observed that the biomass holding capacity of the sponge carrier was better than for the plastic carrier. The hybridization of the MBR and MBBR with sponge carriers was deemed the best method to improve the performance of wastewater treatment [9]. Thus, the hybrid MBBMR has a high effluent production, longer operational duration (48 days), and less sludge generation (44.2 kg dry sludge 106 L^−1^ treated wastewater) [9].

### 3.4. The Biological Methods

The MBR is a technique in which the solid-liquid separation is done through a membrane. MBR is an innovative technology in the field of wastewater treatment. This technology has been utilized at the commercial level for more than 30 years. Besides a high effluent quality, less sludge production, and smaller footprint, a major drawback of MBR is membrane biofouling which limits the implementation of this technology [36]. In the 1970s, QS was first proposed as a mechanism for the coordinated expression of a phenotype, such as bioluminescence, at the population level [37]. QS is a process in which the bacterial cells produce signal molecules (autoinducers) for their intercellular communication. Once this communication occurs, it induces group behaviours such as biofilm formation on the membrane surface [38]. It has been demonstrated that the QS controls the gene expression that mediates some bacterial activities, including the production of soluble microbial products (SMP) and EPS, the secretion of exocellular enzymes, and the development of biofilms [15,39].

Recent approaches have demonstrated the benefits of novel biological strategies based on QS against biofouling. One of these unique methods is QQ, reported as being able to successfully reduce biofouling by reducing QS and focusing on inhibiting the production of N-Acyl Homoserine Lactones (AHLs) [16]. Furthermore, the TMP (proportional to fouling resistance) and the AHL levels increased in a similar pattern, which illustrated the close relationship between the biofouling and QS activity. The conclusion was strengthened by the fact that the suspended biomass in the reactor did not contain significant concentrations of the AHLs. Consequently, this particular study concluded that the AHLs found in the membrane biofilms were produced by the microbial community that grew on the membranes [37].

To control the concentration of AHLs and delay the TMP profile, an advanced idea of QQ has been introduced via the use of acylase or lactonase enzymes, i.e., AHLs degrading enzymes, or by incorporating QQ bacteria producing these enzymes. *Rhodococcus* sp. BH4 entrapped in sodium alginate beads and added into the MBR (with a working volume of 1.6 L for 18 days) generated a significant reduction in the concentration of AHLs [40].

Many researchers have widely applied and studied various QQ media (vessel, bead, cylinder, hollow cylinder, sheet, etc.) [16,41]. According to them, the surface area of the QQ media is a key factor in increasing the activity of the process. The study of Lee and colleagues revealed that hollow cylinders of QQ media were more effective in delaying the biofouling because of their larger surface areas [21,32]. Instead of QQ enzymes, *Rhodococcus* sp. BH4 is a more common solution for MBRs. Sodium alginate (SA) and polyvinyl alcohol (PVA) solutions were used to prepare QQ sheets containing different amounts of *Rhodococcus* sp. BH4 (50 mg, 75 mg, or 100 mg per sheet).

QQ vessels made of microporous hollow fiber membranes, with QQ bacteria narrowed and sealed on the lumen side, were developed and applied to MBRs. In lab scale equipment, it has been shown that the QQ vessel significantly reduced the membrane fouling, although only minimum aeration was provided for the membrane scouring. This demonstrates the possibility of lowering the operational energy consumption for aeration and filtration in the MBR. Throughout the operation (100 days), the microbial vessel consistently sustained its QQ activity [42,43]. The QQ beads were made up of PVA that were more stable for entrapping living cells. Therefore, good quality QQ cell entrapping beads (QQ-CEBs) were made by PVA and were utilized for biofouling control and their effect on the MBR performance was investigated [16]. PVA with a polymerization degree of 2270 was used to create excellent quality PVA-alginate beads. It was found that 1% SA and 8% PVA were the best concentrations for producing PVA-alginate beads. A positive effect on the inner structure of the beads was found to keep the temperature of the first cross-linking solution at 40 °C, the same as the temperature of the PVA alginate mixture solution. This temperature increase had no effect on the viability of the QQ bacteria. SEM images confirmed the immobilization of *Rhodococcus* sp. BH4 within the beads. It was also discovered that QQ-CEBs (QQ bacteria-entrapped PVA-alginate beads) had a high potential for improving the performance of the MBR through a combination of biological and physical cleaning [26].

The QQ technology has been used successfully at a lab-scale in MBRs to treat synthetic municipal wastewater, as well as at a pilot-scale for the treatment of real municipal wastewater. So far, there has been no report of QQ technology being used in MBR systems for industrial wastewater treatment [36].

## 4. Model-Based Anti-Biofouling Strategies

As illustrated in the previous chapters, biofouling is an important challenge in the operation of MBRs, often referred to as the “Achilles heel” [44] of the membrane technology. Being an extremely complex phenomenon, the influence of the various parameters, whether physical or biological, on the development of fouling is not completely understood [45]. To enhance the performance of such systems, automatic solutions that can regularly monitor and adjust parameters during the operation (e.g., flow rate, pressure, pH, cleaning methods and procedures, etc.) are integrated with the MBRs, either online or in real-time [46,47,48].

Mathematical modelling and process simulation tools are extensively used in many process industries to assist the decision-making in the analysis, design, optimization, and control of physical, chemical, and/or biological systems. The rise of networking and digitalization in the context of Industry 4.0, as well as the development of communication technologies, significantly increase the amount of data available on the operation of MBRs [40]. The power of computing technologies has grown significantly and provides solutions to solving increasingly complex industrial problems.

To provide effective biofouling remedies, some of the key areas that require improvement relate to [44] are:

1. Understanding of the specific processes that govern biofilm formation,
2. implementation of pre-treatment techniques that can successfully prevent biofilm formation,
3. monitoring biofouling to enable proactive and effective membrane cleaning and maintenance.

Advancing digital technologies, combined with novel Big Data analytics and optimization techniques, offer great opportunities for creating intelligent systems that can address effectively the challenges of MBR biofouling. The following sections provide an overview of the current approaches that can support the understanding of biofouling in MBRs and provide improved anti-biofouling strategies.

### 4.1. Model-Based Experimental Analysis and Design

Mathematical models are classified into three main categories:

1. Mechanistic (deterministic, white-box), which provide a mathematical representation of the process or system based on physical, chemical, and/or biological understandings.
2. Empirical (black-box, data-based), developed based on experimental data, without assuming relationships between the inputs and the outputs of the process/system.
3. Semi-empirical (gray-box), which consider both mechanistic and empirical characteristics, with the conservation equations based on deterministic understandings and the rate laws on data.

An overview of the various solutions available for the modelling of MBR biofouling will be presented in the next sections, together with their challenges/limitations as well as recommendations for future work.

#### 4.1.1. Mechanistic Tools

Experimental observation of different mechanisms that influence biofouling and the analysis of information on the effects resulted from the periodic cleaning operations of the membrane modules, required to properly manage MBRs [44], can be supplemented by the utilization of mathematical tools.

Biological process modelling approaches are essential in the understanding of MBR systems. Although the basic aspects of biofilm formation and growth are similar in membrane biofouling as in other natural and engineered biological systems, factors such as pressure-driven water and the solute transport phenomenon have a strong influence on the kinetic mechanisms [49]. Furthermore, as the membrane comes in direct contact with the mixed liquor, the physicochemical characteristic of this active biological suspension has an influence on the membrane separation process [50]. Finally, last but not least, membrane cleaning involves mechanical, chemical, or biological actions, targeting specific parameters [51,52]. Thus, modelling such systems requires an understanding of the biofouling process not only at various scales (from the molecular behaviour of the microorganisms at the cellular level to the meso-scale of the membrane separation and the macroscale of the reactor or the process level), but a multiphysics-type of approach to consider all these heterogeneous aspects.

One of the first type of models developed to describe MBRs is based on the activated sludge models [53,54,55], which generally focus on the representation of the biological processes that take place inside the MBR. Since 1987, when the first version (ASM 1) had been introduced for the design and operation of ammonia and organic matter removal in biological wastewater processes, two others, ASM 2 and ASM 3 have been proposed in order to address the removal of phosphorus. Furthermore, several variations—ASM2d, ASM3 + BioP, ASM2d + TUD [56]—have been proposed since 1997 to fix shortcomings of the original ASM 1 model. A main limitation of the ASM models is that their applicability should be carefully verified due to the utilization of uncorroborated assumptions such as the use of linear kinetic rates or the consideration of constant variables, e.g., temperature, pH, kinetic variables, biomass composition, foulants concentration, model parameters in the rate expressions, or parameters related to nitrification [57].

The presence of soluble microbial products (SMPs) has been recognized as one of the main factors leading to membrane fouling [58]. In order to improve the understanding and the representation of the individual foulants, starting with the late 1980s, mathematical models have been developed to include the contribution of the SMPs into the MBR bioprocesses either individually [59,60] or as an extension of the ASMs [61,62,63]. This stage represents the inception of the hybrid biological models for MBRs, which continues with the inclusion of further aspects such as processes associated with EPS [64,65,66]. These new representations enhance the performance of the ASMs in the modelling of the biomass kinetics inside the MBR without the need for calibration with experimental data. However, the addition of SMP components and other variables generally results in complex models with many variables which can make their validation difficult due to over-parameterization [58].

**Figure 3:**
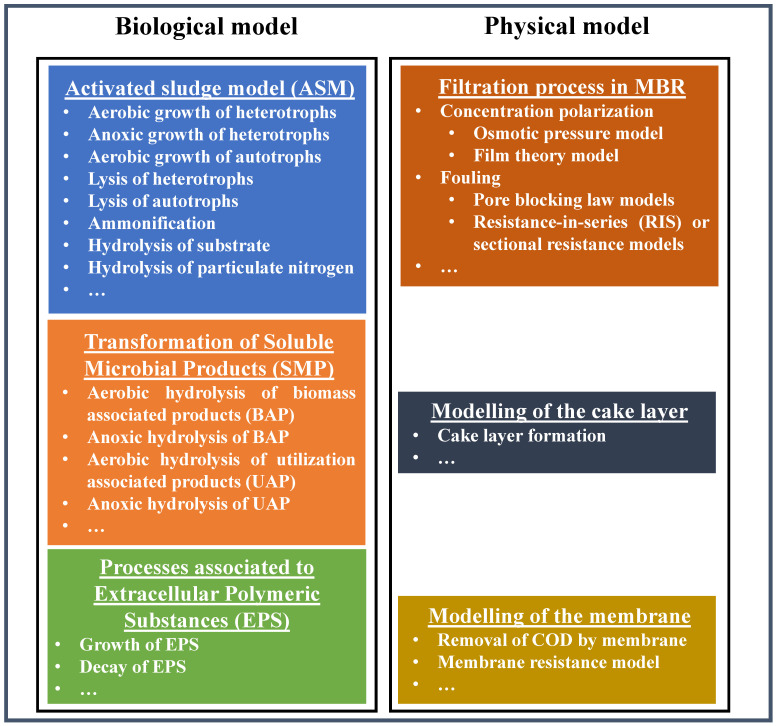
A conceptual framework for the mechanistic modelling of the biological processes inside the MBR (adapted from [56,49]).

Furthermore, in modelling the MBR fouling process, the effect of the parameters that influence the filtration process mechanism (e.g., flow rate, transmembrane pressure) cannot be neglected [67]. These are part of the physical model module in the representation of the biofouling inside MBR (Figure 3) and require quantitative expressions to represent the filtration performance as a function of the hydrodynamic and permeate properties of the membrane [8,68,69,70,71].

Thus, different phenomena involved in biofouling (e.g., cake formation, complete or partial pore blocking, etc.) must be taken into account for the development of accurate and reliable MBR models, in an integrated approach that considers both biological and physical processes of the system. Each of these two separate modules are able to improve the understanding of the various MBR sub-processes. Furthermore, as they are strongly interconnected and interact with each other, their integration should be always considered to ensure the optimal condition for the process and to enhance the understanding of the development of fouling.

Numerous studies have been devoted to the development and implementation of such integrated models for the prediction of the MBR behaviour [72,73,74,75].

It is obvious that the development of such models significantly increases their complexity, yet they are the best approach for providing a clear understanding of the processes that take place inside the MBRs. However, uncertainties are present during all stages, from the definition of the process and data collection, model development, implementation, and validation, to the model simulation and the interpretation of the results. Additionally, uncertainty in understanding the underlying physical, chemical, and biological phenomena can reduce the accuracy of the resulting models. Further research in various MBR applications is required to balance the complexity and nonlinearity and reduce the uncertainty of the resulting integrated models. Moreover, reducing the number of variables should be considered to improve the accuracy of the models for efficient prediction of the performance of the MBRs. Data-based approaches can support achieving this objective.

#### 4.1.2. Machine Learning Approaches

In addition to the mechanistic models discussed in the previous section, the fourth industrial revolution provides novel technologies to advance the understanding of MBR biofouling, as well as to play an important role in optimizing the process parameters and its operations [48].

The accurate representation of the MBR at multiple-scales strongly relies on the model structure and its parameter values, which have to be fine-tuned to correspond to the real process. To this end, experimental data are required to estimate and validate the resulting models.

Novel monitoring techniques [47,76,77,78,79] based on innovative technologies such as smart sensors and the Internet of Things, as well as the continuous improvement and miniaturization of sensors and actuators results in the possibility of having every element of the MBR connected with such a device that is capable of gathering and exchanging accurate and reliable data on its state, often in real-time.

Big Data analytics and tools based on Artificial Intelligence and Machine Learning provide reliable and flexible means for improving MBR processes by increasing the accuracy and sensitivity of the resulting models through the analysis of the huge amounts of data that can be collected at various plant scales.

Models based on neural networks are some of the oldest and most commonly applied in MBR systems [30,80,81], due to their ability to produce multiple inputs and multiple outputs (MIMO) models, to work with noisy and incomplete data, to utilize complicated non-linear functions with high accuracy, to update/train the resulted models with new data, as well as due to their ability to reduce computational times [82]. Major limitations of the classical algorithms (e.g., back-propagation) utilized in the training of artificial neutral network (ANN) models are related to their slow rate of convergence, stopping in a local optimum, and their use of trial-and-error approaches for determining the number of neurons in the hidden layers.

To overcome these challenges, hybrid optimization techniques based on genetic algorithms [83,84] and particle swarm optimization [57,85] have been implemented. Although with some advantages versus conventional ANN algorithms, these approaches suffer from issues such as a slow rate of convergence, high computational cost due to their complex structure, or the requirement of considerable data sizes [57]. Furthermore, in many cases, they also fall under local optima in high-dimensional spaces.

Thus, novel approaches are required for the training of neural networks that incorporate solutions to selectively train neurons by, i.e., identifying their importance through neural interpretation diagrams [86] or relevance scores [87] have been proposed. While they focus exclusively on the visualization of the neural importance, they are either too simplistic or highly complicated, requiring further development for efficient applications. Consequently, further research is required into the development of approaches with moderate complexity that enable the evaluation of the neural importance for the purpose of the selective tuning of the network and applying and tuning them towards the use for applications in MBR biofouling.

Due to the complex structure and the highly interconnected nature of the phenomena taking place inside the MBR, techniques that combine deterministic tools (i.e., Artificial Intelligence and Machine Learning) to inform mechanistic models have the potential to predict the realistic behaviour of such systems. Data-based approaches can provide the relationships between the various factors that influence the process, which can then be used in the multiscale models to enhance their accuracy.

### 4.2. Optimization-Based Strategies

Improving the understanding of the insights of the operation of MBRs provides further pathways towards the mitigation of biofouling. Models can be used for the development of strategies for the scheduling of cleaning actions of the membranes in maintenance optimization models. Such models quantify both the costs and benefits of the anti-biofouling measures, trying to achieve an optimum balance while taking into account all the relevant operational constraints.

State-of-the-art approaches for membrane cleaning scheduling are generally focused on approaches based on Mixed-Integer Nonlinear Programming (MINLP) formulations of the process [88]. However, the use of MINLP methods lead to a combinatorial problem that is often computationally expensive and is not able to accurately capture the behaviour of the system under investigation [89,90].

Recent work in the area of performance decaying processes such as heat exchanger networks [91], catalytic reactors [92], and reverse osmosis [93] utilizes the optimal control problem (OCP) theory for the reformulation of the cleaning scheduling problem (a nonlinear non-convex dynamic optimization problem with binary variables) as a multistage integer nonlinear optimal control problem (MSINOCP). The main advantage is that in this reformulation, the decisions variables for cleaning are present linearly in the optimization model, displaying a bang-bang behaviour (i.e., the control variable takes values at either bounds of the feasible region). Another advantage of this solution scheme is that it can handle successfully uncertain parameters of the model [94]. Finally, it can incorporate a realistic dynamic behaviour of the membrane (exponential decay of the membrane permeability over time, age of the membrane, membrane flux recovery, concentration polarization, etc.) and it produces more robust maintenance schedules, with less cleaning that could damage the membrane.

## 5. Conclusions

Biofouling is still a great challenge in MBR operation. Novel approaches such as QQ seem to be very promising to prevent initializing the primary fouling stages. Currently, it is well known that the cell-to-cell communication of bacteria in the sludge is one major reason responsible for biofouling. This review introduced some of the novel approaches for the prevention of the initial primary fouling stages. Of these, QQ is identified as the most promising, with two bacterial strains, *Rhodococcus* sp. BH4 and *Pseudomonas putida*, being very effective in the disruption of QS. In the future it is expected that several other novel strains will be identified in supporting QQ and improve the response to MBR biofouling.

Besides QS, another important challenge is the limited knowledge on biofouling in MBRs due to its complexity related to the multiscale and multiphysics aspects of the phenomena involved. It is shown that an accurate mathematical representation of the MBR processes can provide a detailed understanding of the biofouling process. Current modelling techniques based on hybrid approaches combining mechanistic and data-based approaches, as well as optimization techniques, can support the development of reliable models that can support the decision-making for such a complex system and provide users the right tools for efficient and cost-effective operation of the MBR.

Future research should focus on identifying the most efficient ways of understanding the transport phenomena that occur inside the reactor elements (e.g., membrane module, biological and fouling processes, etc.), as well as the integrated modelling of MBRs by utilizing systematic approaches for the analysis of the system and implementation of the resulting models in process modelling and simulation tools such as Aspen Plus or gPROMS.

## Figures and Tables

**Table 1:** The factors affecting membrane fouling in MBR.

Membrane Characteristics	Operational Conditions	Feed and Biomass Characteristics
Material type	Operating mode	Mixed liquor suspended solids
	Aeration rate	Sludge apparent viscosity
Water affinity	Solid retention time	Extracellular polymeric substances
Surface roughness	Hydraulic retention time	Floc size
Surface charge	Temperature	Alkalinity and pH
Pore size	Organic loading rate	Salinity

## Data Availability

The data that support the findings of this review are available on request from the corresponding author.

## References

[1] Habiba U., Taj L., Farid M., Anwar-ul-haq M., Sharif N., Farheen H., Sharif N. (2013). Quality Analysis of Ground Water Resources of Paharang Drain Faisalabad, Pakistan. Int. J. Sci. Environ. Technol..

[2] Abbas T., Majeed A.D. (2016). Applicability of MBR Technology for Decentralized Municipal Wastewater Treatment in Iraq.

[3] Maqbool N. (2022). Water Crisis in Pakistan: Manifestation, Causes and the Way Forward.

[4] Hussain A. (2018). Per Capita Water Availability in Pakistan Comes to Dangerously Low Level. Pakistan Today. https://archive.pakistantoday.com.pk/2018/03/22/per-capita-water-availability-in-pakistan-comes-to-dangerously-low-level/.

[5] Leyva-Díaz J.C., Martín-Pascual J., González-López J., Hontoria E., Poyatos J.M. (2013). Effects of scale-up on a hybrid moving bed biofilm reactor–membrane bioreactor for treating urban wastewater. Chem. Eng. Sci..

[6] Leyva-Díaz J.C., Martín-Pascual J., Muñío M.M., González-López J., Hontoria E., Poyatos J.M. (2014). Comparative kinetics of hybrid and pure moving bed reactor-membrane bioreactors. Ecol. Eng..

[7] Cui Y., Gao H., Yu R., Gao L., Zhan M. (2021). Biological-based control strategies for MBR membrane biofouling: A review. Water Sci. Technol..

[8] Du X., Shi Y., Jegatheesan V., Haq I.U. (2020). A review on the mechanism, impacts and control methods of membrane fouling in MBR system. Membranes.

[9] Sohail N., Ahmed S., Chung S., Nawaz M.S. (2020). Performance comparison of three different reactors (MBBR, MBR and MBBMR) for municipal wastewater treatment. Desalin. Water Treat..

[10] Karim M.A., Mark J.L. (2017). A Preliminary Comparative Analysis of MBR and CAS Wastewater Treatment Systems. Int. Water Wastewater Treat..

[11] Gao M., Zou X., Dang X., Mohammed A.N., Yang S., Zhou Y., Yao Y., Guo H., Liu Y. (2023). Exploring interactions between quorum sensing communication and microbial development in anammox membrane bioreactor. J. Environ. Chem. Eng..

[12] Cui Z., Ngo H.H., Cheng Z., Guo W., Meng X., Jia H., Wang J. (2020). Hysteresis effect on backwashing process in a submerged hollow fiber membrane bioreactor (MBR) applied to membrane fouling mitigation. Bioresour. Technol..

[13] Jiang C.K., Tang X., Tan H., Feng F., Xu Z.M., Mahmood Q., Zeng W., Min X.B., Tang C.J. (2019). Effect of scrubbing by NaClO backwashing on membrane fouling in anammox MBR. Sci. Total Environ..

[14] Fortuanto L., Ranieri L., Naddeo V., Leiknes T. (2020). Fouling control in a gravity-driven membranes (GDM) bioreactor treating primary wastewater by using relaxation and/or air scouring. J. Membr. Sci..

[15] Syafiuddin A., Boopathy R., Mehmood M.A. (2021). Recent advances on bacterial quorum quenching as an effective strategy to control biofouling in membrane bioreactors. Bioresour. Technol..

[16] Ahmed S., Chung S., Sohail N., Qazi I.A., Justin A. (2020). Application of cell entrapping beads for Quorum Quenching technique in submerged membrane bioreactor. Water Sci. Technol..

[17] Singh D., Satpute S.K., Ranga P., Saharan B.S., Tripathi N.M., Aseri G.K., Sharma D., Joshi S. (2022). Biofouling in membrane bioreactors: Mechanism, interactions and possible mitigation using biosurfactants. Appl. Biochem. Biotechnol..

[18] Xiong J., Zuo S., Liao W., Chen Z. (2019). Model-based evaluation of fouling mechanisms in powdered activated carbon/membrane bioreactor system. Water Sci. Technol..

[19] Leyva-Díaz J.C., Munío M.M., Gonzalez-López J., Poyatos M.J. (2016). Anaerobic/anoxic/oxic configuration in hybrid moving bed biofilm reactor-membrane bioreactor for nutrient removal from municipal wastewater. Ecol. Eng..

[20] Iorhemen O.T., Hamza R.A., Tay J.H. (2016). Membrane bioreactor (MBR) technology for wastewater treatment and reclamation: Membrane fouling. Membranes.

[21] Huyskens C., Brauns E., Van Hoof E., De Wever H. (2008). A new method for the evaluation of the reversible and irreversible fouling propensity of MBR mixed liquor. J. Membr. Sci..

[22] Guo W., Ngo H.H., Li J. (2012). A mini-review on membrane fouling. Bioresour. Technol..

[23] Skinner S.J., Stickland A.D., Scales P.J. (2018). Predicting transmembrane pressure rise from biofouling layer compressibility and permeability. Chem. Eng. Technol..

[24] Wang Z., Ma J., Tang C.Y., Kimura K., Wang Q., Han X. (2014). Membrane cleaning in membrane bioreactors: A review. J. Membr. Sci..

[25] Wu J., Le-Clech P., Stuetz R.M., Fane A.G., Chen V. (2008). Effects of relaxation and backwashing conditions on fouling in membrane bioreactor. J. Membr. Sci..

[26] Islam Z.U., Rose J., Ahmed S., Chung S. (2020). Quorum Quenching Cell Entrapping Bead by Polyvinyl Alcohol Method for Biofouling Mitigation in Lab-scale MBR. J. Eng. Sci..

[27] Chang H., Liang H., Qu F., Liu B., Yu H., Du X., Li G., Snyder S.A. (2017). Hydraulic backwashing for low-pressure membranes in drinking water treatment: A review. J. Membr. Sci..

[28] Krzeminski P., Leverette L., Malamis S., Katsou E. (2017). Membrane bioreactors—A review on recent developments in energy reduction, fouling control, novel configurations, LCA and market prospects. J. Membr. Sci..

[29] Ayub M., Saeed N., Chung S., Nawaz M.S., Ghaffour N. (2020). Physical and economical evaluation of laboratory-scale membrane bioreactor by long-term relative cost–benefit analysis. J. Water Reuse Desalin..

[30] Cai W., Liu J., Zhu X., Zhang X., Liu Y. (2017). Fate of dissolved organic matter and byproducts generated from on-line chemical cleaning with sodium hypochlorite in MBR. J. Chem. Eng..

[31] Wang S., Chew J.W., Liu Y. (2020). An environmentally sustainable approach for online chemical cleaning of MBR with activated peroxymonosulfate. J. Membr. Sci..

[32] Robescu D., Calin A., Robescu D., Nasaramba B. Simulation of attached growth biological wastewater treatment process in the mobile bed biofilm reactor. Proceedings of the 10th WSEAS International Conference on Mathematic and Computers in Biology and Chemistry.

[33] Khan S.J., Ilyas S., Javid S., Visvanathan C., Jegatheesan V. (2011). Performance of suspended and attached growth MBR systems in treating high strength synthetic wastewater. Bioresour. Technol..

[34] Mannina G., Capodici M., Cosenza A., Cina P., Di Trapani D., Puglia A.M., Ekama G.A. (2017). Bacterial community structure and removal performances in IFAS-MBRs: A pilot plant case study. J. Environ. Manag..

[35] Guo W., Ngo H.H., Vigneswaran S., Xing W., Goteti P. (2008). A Novel Sponge-Submerged Membrane Bioreactor (SSMBR) for Wastewater Treatment and Reuse. Sep. Sci. Technol..

[36] Liu J., Sun F., Zhang P., Zhou Y. (2021). Integrated powdered activated carbon and quorum quenching strategy for biofouling control in industrial wastewater membrane bioreactor. J. Clean. Prod..

[37] Siddiqui M.F., Rzechowicz M., Harvey W., Zularisam A.W., Anthony G.F. (2015). Quorum sensing based membrane biofouling control for water treatment: A review. J. Water Process Eng..

[38] Weerasekara N.A., Choo K.H., Lee C.H. (2014). Hybridization of physical cleaning and quorum quenching to minimize membrane biofouling and energy consumption in a membrane bioreactor. Water Res..

[39] Jiang W., Xia S., Liang J., Zhang Z., Hermanowicz S.W. (2013). Effect of quorum quenching on the reactor performance, biofouling and biomass characteristics in membrane bioreactors. Water Res..

[40] Maqbool T., Khan S.J., Waheed H., Lee C.H., Hashmi I., Iqbal H. (2015). Membrane biofouling retardation and improved sludge characteristics using quorum quenching bacteria in submerged membrane bioreactor. J. Membr. Sci..

[41] Oh H.S., Lee C.H. (2018). Origin and evolution of quorum quenching technology for biofouling control in MBRs for wastewater treatment. J. Membr. Sci..

[42] Iqbal T., Lee K., Lee C.H., Choo K.H. (2018). Effective quorum quenching bacteria dose for anti-fouling strategy in membrane bioreactors utilizing fixed-sheet media. J. Membr. Sci..

[43] Huang J., Gu Y., Zeng G., Yang Y., Ouyang Y., Shi L., Shi Y., Yi K. (2018). Control of indigenous quorum quenching bacteria on membrane biofouling in a short-period MBR. Bioresour. Technol..

[44] Hoek E.M.V., Weigand T.W., Edalat A. (2022). Reverse osmosis membrane biofouling: Causes, consequences and countermeasures. npj Clean Water.

[45] Di Bella G., Di Trapani D. (2019). A brief review on the resistance-in-series model in membrane bioreactors (MBRs). Membranes.

[46] Fortunato L., Pathak N., Rehman Z.U., Shon H., Leiknes T. (2018). Real-time monitoring of membrane fouling development during early stages of activated sludge membrane bioreactor operation. Process Saf. Environ. Prot..

[47] Santos A.V., Lin A.R.A., Amaral M.C.S., Oliveira S.M.A.C. (2021). Improving control of membrane fouling on membrane bioreactors: A data-driven approach. J. Chem. Eng..

[48] Viet N.D., Jang A. (2023). Comparative mathematical and data-driven models for simulating the performance of forward osmosis membrane under different draw solutions. Desalination.

[49] Mitra S., Murthy G.S. (2022). Bioreactor control systems in the biopharmaceutical industry: A critical perspective. Syst. Microbiol. Biomanuf..

[50] AlSawaftah N., Abuwatfa W., Darwish N., Husseini G.A. (2022). A review on membrane biofouling: Prediction, characterization, and mitigation. Membranes.

[51] Patsios S.I., Karabalas A.J. (2010). A review of modelling bioprocesses in membrane bioreactors (MBR) with emphasis on membrane fouling predictions. Desalin. Water Treat..

[52] Maddah H., Chogle A. (2017). Biofouling in reverse osmosis: Phenomena, monitoring, controlling and remediation. Appl. Water Sci..

[53] Gizer G., Önal U., Ram M., Sahiner N. (2023). Biofouling and mitigation methods: A review. Biointerface Res. Appl. Chem..

[54] Fenu A., Guglielmi G., Jimenez J., Sperandio M., Saroj J., Lesjean B., Brepols C., Thoeye C., Nopens I. (2010). Activated sludge model (ASM) based modeling of membrane bioreactor (MBR) processes: A critical review with special regard to MBR specificities. Water Res..

[55] Tenore A., Vieira J., Frunzo L., Luongo V., Fabbricino M. (2020). Calibraton and validation of an activated sludge model for membrane bioreactor wastewater treatment plants. Environ. Technol..

[56] Mannina G., Cosenza A., Rebouças T.F. (2020). A plant-wide modelling comparison between membrane bioreactors and conventional activated sludge. Bioresour. Technol..

[57] Hauduc H., Rieger L., Oehmen A., Van Loosdrecht M.C.M., Comeau Y., Heduit A., Vanrolleghem P.A., Gillot S. (2012). Critical review of activated sludge modelling: State of processs knowledge, modelling concepts, and limitations. Biotechnol. Bioeng..

[58] Hamedi H., Mohammadzadeh O., Rasouli S., Zendehboudi S. (2021). A critical review of biomass kinetics and membrane filtration models for membrane bioreactor systems. J. Environ. Chem. Eng..

[59] Menniti A., Morgenroth E. (2010). Mechanisms of SMP production in membrane bioreactors: Choosing an appropriate model structure. Water Res..

[60] Shi Y., Huang J., Zeng G., Gu Y., Hu Y., Tang B., Zhou J., Yang Y., Shi L. (2018). Evaluation of soluble microbial products (SMP) on membrane fouling in membrane bioreactors (MBRs) at the fractional and overall level: A review. Rev. Environ. Sci. Biotechnol..

[61] Zuthi M.F.R., Ngo H.H., Guo W.S., Zhang J., Liang S. (2013). A review towards finding a simpliefied approach for modelling the kinetics of soluble microbial products (SMP) in an integrated mathematical model of membrane bioreactor (MBR). Int. Biodeter. Biodegr..

[62] Benyahia B., Sari T., Cherki B., Harmand J. (2013). Anaerobic membrane bioreactor modelling in the presence of soluble microbial products (SMP)—The Anaerobic model AM2b. J. Chem. Eng..

[63] Singh R.P., Fu D., Yang J., Xiong J. (2019). Operational performance and biofoulants in a dynamic membrane bioreactor. Bioresour. Technol..

[64] Nadeem K., Alliet M., Plana Q., Bernier J., Azimi S., Rocher V., Albasi C. (2022). Modelling, simulation and control of biological and chemical P-removal processes for membrane bioreactors (MBRs) from lab to full-scale applications: State of the art. Sci. Total Environ..

[65] Janus T. (2014). Integrated mathematical model of a MBR reactor including biopolymer kinetics and membrane fouling. Procedia Eng..

[66] Janus T., Ulanicki B. (2010). Modelling SMP and EPS formation and degradation kinetics with an extended ASM3 model. Desalination.

[67] Lindamulla L.M.L.K.B., Jegatheesan V., Jinadasa K.B.S.N., Nanayakkara K.G.N., Othman M.Z. (2021). Integrated mathematical model to simulate the performance of a membrane bioreactor. Chemosphere.

[68] Teng J., Zhang M., Leung K.T., Chen J., Hong H., Lin H., Liao B.Q. (2019). A unified thermodynamic mechanism underlying fouling behaviours of soluble microbial products (SMPs) in a membrane bioreactor. Water Res..

[69] Wu M., Zhang M., Shen L., Wang X., Ying D., Lin H., Li R., Xu Y., Hong H. (2023). High propensity of membrane fouling and the underlying mechanisms in a membrane bioreactor during occurrence of sludge bulking. Water Res..

[70] Teng J., Zhang H., Tang C., Lin H. (2021). Novel molecular insights into forward osmosis membrane fouling affected by reverse diffusion of draw solutions based on thermodynamic mechanisms. J. Membr. Sci..

[71] Teng J., Zhang H., Lin H., Wang J., Meng F., Wang Y., Lu M. (2022). Synergistic fouling behaviours and thermodynamic mechanisms of proteins and polysaccharides in forward osmosis: The unique role of reverse solute diffusion. Desalination.

[72] Long Y., You X., Chen Y., Hong H., Liao B.Q., Lin H. (2020). Filtration behaviours and fouling mechanisms of ultrafiltration processes with polyacrylamide flocculation for water treatment. Sci. Total Environ..

[73] Zuthi M.F.R., Ngo H.H., Guo W.S. (2012). Modelling bioprocesses and membrane fouling in membrane bioreactor (MBR): A review towards finding an integrated model framework. Bioresour. Technol..

[74] Zuthi M.F.R., Guo W., Ngo H.H., Nghiem D.L., Hai F.I., Xia S., Li J., Li J., Liu Y. (2017). New and practical mathematical model of membrane fouling in an aerobic submerged membrane bioreactor. Bioresour. Technol..

[75] Brepols C., Comas J., Harmand J., Robles A., Rodriguez-Roda I., Ruano M.V., Smets I., Mannina G. (2020). Position paper—Progress towards standards in integrated (aerobic) MBR modelling. Water Sci. Technol..

[76] Gonzalez-Hernandez Y., Jauregui-Haza U.J. (2021). Improved integrated dynamic model for the simulation of submerged membrane bioreactors for urban and hospital wastewater treatment. J. Membr. Sci..

[77] Yu J., Xiao K., Xu H., Qi T., Li Y., Tan J., Wen X., Huang X. (2021). Spectroscopic sensing of membrane fouling potential in a long-term running anaerobic membrane bioreactor. J. Chem. Eng..

[78] Han H., Zhang S., Qiao J., Wang X. (2018). An intelligent detecting system for permeability prediction of MBR. Water Sci. Technol..

[79] Ba-Alawi A.H., Nam K., Heo S., Woo T., Aamer H., Yoo C. (2023). Explainable multisensory fusion-based automatic reconciliation and imputation of faulty and missing data in membrane bioreactor plants for fouling alleviation and energy saving. Chem. Eng. J..

[80] Zhang X., Cheng X., Reng J., Ma X., Liu Q., Yao P., Ngo H.H., Nghiem L.D. (2021). UV assisted backwashing for fouling control in membrane bioreactor operation. J. Membr. Sci..

[81] Yang H., Yu X., Liu J., Tang Z., Huang T., Wang Z., Zhong Y., Long Z., Wang L. (2022). A concise review of theoretical models and numerical simulations of membrane fouling. Water.

[82] Zhuang L., Tang B., Bin L., Li P., Huang S., Fu F. (2021). Performance prediction of an internal-circulation membrane bioreactor basd on models comparison and data features analysis. Biochem. Eng. J..

[83] Jawad J., Hawari A.H., Zaidi S.J. (2021). Artificial neural network modelling of wastewater treatment and desalination using membrane processes: A review. Biochem. Eng. J..

[84] Li C., Yang Z., Yan H., Wang T. (2014). The application and research of the GA-BP neural network algorithm in the MBR membrane fouling. Abstr. Appl. Anal..

[85] Yao J., Wu Z., Liu Y., Zheng X., Zhang H., Dong R., Qiao W. (2022). Predicting membrane fouling in a high solid AnMBR treating OFMSW leachate through a genetic algorithm and the optimization of a BP neural network model. J. Environ. Manag..

[86] Yusuf Z., Wahab N.A., Sudin S. (2019). Soft computing techniques in modelling of membrane filtration system: A review. Desalin. Water Treat..

[87] Ozesmi S.L., Ozesmi U. (1999). An artificial neural network approach to spatial habitat modelling with interspecific interaction. Ecol. Modell..

[88] Montavon G., Binder A., Lapushckin S., Samek W., Muller K.R. (2019). Layer-wise relevance propagation: An overview. Explainable AI: Interpreting.

[89] Ko D. (2018). Conceptual design optimization of an integrated membrane bioreactor system for wastewater treatment. Chem. Eng. Res. Des..

[90] Puchongkawarin C., Gomez-Mont C., Stuckey D.C., Chachuat B. (2015). Optimization-based methodology for the development of wastewater facilities for energy and nutrient recovery. Chemosphere.

[91] Aboagye E.A., Burnham S.M., Dailey J., Zia R., Tran C., Desai M., Yenkie K.M. (2021). Systematic design, optimization, and sustainability assessment for generation of efficient wastewater treatment networks. Water.

[92] Al Ismaili R., Lee M.W., Wilson D.I., Vassiliadis V.S. (2018). Heat exchanger network cleaning scheduling: From optimal control to mixed-integer decision making. Comput. Chem. Eng..

[93] Adloor S.D., Pons T., Vassiliadis V.S. (2020). An optimal control approach to scheduling and production in a process using decaying catalysts. Comput. Chem. Eng..

[94] Mappas V., Vassiliadis V.S., Dorneanu B., Routh A.F., Arellano-Garcia H. (2022). Maintenance scheduling optimisation of reverse osmosis networks (RONs) via a multistage optimal control reformulation. Desalination.

